# Enhancing cell growth and antibody production in CHO cells by siRNA knockdown of novel target genes

**DOI:** 10.1186/1753-6561-7-S6-P92

**Published:** 2013-12-04

**Authors:** Sandra Klausing, Oliver Krämer, Thomas Noll

**Affiliations:** 1 Institute of Cell Culture Technology, Bielefeld University, Bielefeld, Germany; 2 Center for Biotechnology (CeBiTec), Bielefeld, Germany

## Background

Seven out of the ten top-selling biopharmaceuticals in 2011 are produced in Chinese Hamster Ovary (CHO) cells [1]. This tremendous commercial interest makes the development and application of strategies for cell line optimization, like gene overexpression or knockdown to enhance cell specific productivity and cellular growth, highly interesting. In this work, we investigated the knockdown effect of novel target genes by siRNA as a powerful tool for CHO cell line engineering.

## Materials and methods

CHO DP-12 cells (clone #1934, ATCC CRL-12445) were used as a model cell line, producing an anti IL-8 antibody. Cultivations were performed in 125 mL shaking flasks at 37 °C, 5% CO_2_, 185 rpm and 5 cm shaker orbit. For fed-batch processes, TCx2D feed supplement (TeutoCell AG) and a predefined feeding regime were applied identically for all cultures. Viable cell densities (vcd) and cell viability were measured by a Cedex Sytem (Innovatis). Monoclonal antibody (mAb) concentrations were determined via HPLC and a protein A column (Life Technologies).

Target genes were chosen based on well-known signaling pathways (e.g. apoptosis, cell cycle or histone modification) as well as from previous results of a CHO cDNA microarray [2]. Mediators of apoptosis Bad and JNK were chosen as target genes for evaluation after knockdown, as well as Set, a protein involved in histone modification. Mcm5 is involved in DNA replication but its regulative role is not completely understood. Finally, knockdown of target gene P (patent pending) was investigated. Short hairpin RNA (shRNA) sequences were designed and cloned into a shRNA expression vector which was stably introduced into CHO DP-12 cells via lentiviral gene delivery. After selection with 5 μg/mL puromycin, successful siRNA-mediated mRNA knockdown (kd) of the target gene was verified by quantitative real-time PCR (qPCR). Transduced cell pools were evaluated in batch and fed-batch shaker cultivations with regard to growth performance and antibody productivity.

## Results

**Figure 1:**
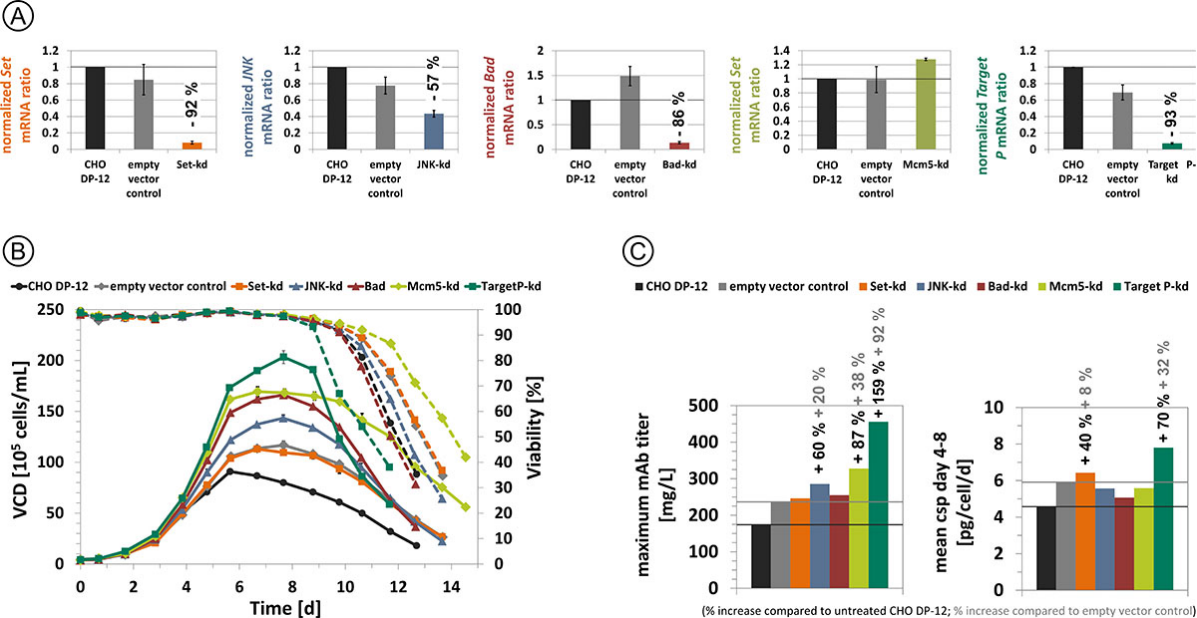
**(A)** Relative mRNA ratio of target genes in cell pools with stable shRNA expression and the empty vector control cell pool compared to untreated CHO DP-12 cells. **(B) **Viable cell density and viability during fed-batch shaker cultivation of cell pools and untreated cells. **(C) **Maximum mAb titer and mean cell specific productivity (csp) between day 4 and 8 for all cultures in fed-batch cultivation.

Through siRNA-mediated RNA interference, a high stable gene knockdown in the cell pools was achieved for target gene Set, JNK, Bad and P. Transcript levels were reduced by 57% (knockdown of JNK) up to 93% (knockdown of P), as shown in Figure 1A. Due to the procedure of lentiviral infection and puromycin selection, a slight variation in transcript levels of some target genes was observed even for an empty vector control cell pool in comparison to untreated CHO DP-12 cells. Unexpectedly, despite genomic integration of Mcm5-targeting shRNA, Mcm5 transcription was found to be up-regulated in two separate measurements of the respective cell pool.

In batch shaker cultivations, all cells with a stable vector integration exhibited higher maximum vcds, compared to the untreated CHO DP-12 culture. Cells with a stable knockdown of apoptosis mediator Bad reached the highest vcd with 121·10^5 ^cells/mL. However, final antibody titers did not exceed the titer of the empty vector control cell pool (data not shown).

Fed-batch shaker cultivation increased maximum cell densities as well as process duration and revealed a strong influence of siRNA mediated gene knockdown (Figure 1B and C). The maximum vcd was increased for cells with stable expression of a shRNA targeting JNK (by 23 %), Bad (by 44 %), Mcm5 (by 45 %) and P (by 74 %) compared to empty vector control cells. In comparison to this control cell pool, maximum mAb titer was higher for cell pools JNK-kd, Mcm5-kd and P-kd. Mean cell specific productivity between day 4 and day 8 of the cultivation was increased in cell pools Set-kd as well as P-kd. The highest mAb titer of 456 mg/L was detected for cells with a stable knockdown of gene P.

## Conclusions

siRNA knockdown of target genes is an effective tool for CHO cell engineering in order to achieve higher viable cell densities and mAb titers. The stable transduction of shRNA targeting Mcm5 resulted in a slight increase of the transcript level, nevertheless, vcd and product titer were enhanced. This effect will be further analyzed. Knockdown of target gene P led to increased vcd in fed-batch cultivation (by 123 %), higher maximum mAb titer (by 159 %) and higher csp between day 4 and 8 (by 70 %), compared to untreated CHO DP-12 cells, which makes this target gene a highly interesting candidate for cell line engineering. Stable transduction with an empty vector also influenced cellular behavior of the control cell pool compared to untreated CHO DP-12 cells. This is likely due to the random integration of the transfer vector and a selection for more robust and faster growing cells during the procedure of lentiviral infection and puromycin selection. Further reasons are under investigation. Single cell clone isolation for the presented cell pools will most likely result in further improvements of viable cell density and product titer.
